# Cooperative integration between HEDGEHOG-GLI signalling and other oncogenic pathways: implications for cancer therapy

**DOI:** 10.1017/erm.2015.3

**Published:** 2015-02-09

**Authors:** Silvia Pandolfi, Barbara Stecca

**Affiliations:** 1 Core Research Laboratory, Istituto Toscano Tumori, Florence, Italy; 2 Department of Oncology, Azienda Ospedaliero-Universitaria Careggi, Florence, Italy

## Abstract

The HEDGEHOG-GLI (HH-GLI) signalling is a key pathway critical in embryonic development, stem cell biology and tissue homeostasis. In recent years, aberrant activation of HH-GLI signalling has been linked to several types of cancer, including those of the skin, brain, lungs, prostate, gastrointestinal tract and blood. HH-GLI signalling is initiated by binding of HH ligands to the transmembrane receptor PATCHED and is mediated by transcriptional effectors that belong to the GLI family, whose activity is finely tuned by a number of molecular interactions and post-translation modifications. Several reports suggest that the activity of the GLI proteins is regulated by several proliferative and oncogenic inputs, in addition or independent of upstream HH signalling. The identification of this complex crosstalk and the understanding of how the major oncogenic signalling pathways interact in cancer is a crucial step towards the establishment of efficient targeted combinatorial treatments. Here we review recent findings on the cooperative integration of HH-GLI signalling with the major oncogenic inputs and we discuss how these cues modulate the activity of the GLI proteins in cancer. We then summarise the latest advances on SMO and GLI inhibitors and alternative approaches to attenuate HH signalling through rational combinatorial therapies.

## Introduction

The HEDGEHOG-GLI (HH-GLI) signalling plays a critical role in embryonic development, stem cell biology and tissue homeostasis, cellular metabolism, synapse formation and nociception (Refs 1, 2, 3, 4, 5). Aberrant activation of the HH signalling has been linked to different aspects of cancer development, from initiation to metastasis (Ref. 6). Canonical HH pathway activation is initiated by the binding of HH ligands to the transmembrane receptor PATCHED (PTCH), which relieves its inhibition on the transmembrane protein SMOOTHENED (SMO). Consequently, active SMO triggers an intracellular signalling cascade leading to the formation of activator forms of the GLI zinc finger transcription factors GLI2 and GLI3, which directly induce GLI1. Both GLI1 and GLI2 act as main mediators of HH signalling in cancer by controlling the expression of target genes.

Recent evidence suggests that GLI proteins can be directly and indirectly modulated by proliferative and oncogenic inputs, in addition or independent of upstream HH signalling. These mechanisms of aberrant, non-canonical HH-GLI pathway activation, apparently without known driver mutations in components of the pathway, have been associated with several types of human cancer (Ref. 7). In this review, we focus on the cooperative interaction between HH-GLI and other oncogenic signalling pathways. We first address the functions and post-translational modifications of the three GLI transcription factors, and the mechanisms that regulate their activity in cancer. We then review latest advances on SMO and GLI inhibitors and discuss approaches to attenuate HH signalling through rational combinatorial therapies.

## Overview of the HEDGEHOG-GLI signalling

The Hh signalling has been initially identified in *Drosophila melanogaster*, where it is required for determining proper embryonic patterning (Ref. 8). Smo protein is conserved and maintains its function in mammals, whereas there are two Ptch proteins in vertebrates. Hh ligand has diversified into Sonic (SHH), Indian (IHH) and Desert (DHH) Hedgehog, and the function of the downstream transcription factor *Cubitus interruptus* (Ci) has evolved into three GLI proteins: GLI1, GLI2 and GLI3. Here we focus on the function and regulation of the three GLI transcription factors and we present only a brief introduction of the key steps and components of vertebrate HH-GLI signalling upstream of GLI.

**Figure 1.**
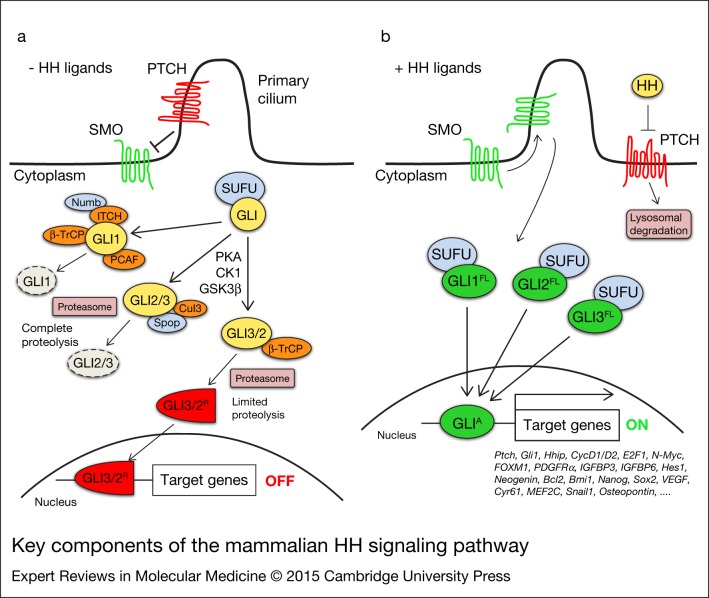
**Key components of the mammalian HH signalling pathway**. In absence of HH ligands (a), PTCH inhibits SMO by preventing its entry into the primary cilium. GLI proteins are phosphorylated by PKA, GSK3β and CK1, which create binding sites for the E3 ubiquitin ligase β-TrCP. GLI3 and, to a lesser extent, GLI2 undergo partial proteasome degradation, leading to the formation of repressor forms (GLI3/2^R^, red), that translocate into the nucleus where they inhibit the transcription of HH target genes. Full-length GLI may also be completely degraded by the proteasome. This process can be mediated by Spop and Cullin 3-based E3 ligase for GLI2 and GLI3, whereas GLI1 can be degraded by β-TrCP, the Numb-activated Itch E3 ubiquitin ligase and by PCAF (see text for details). Upon HH ligand binding (b), PTCH is displaced from the primary cilium, allowing accumulation and activation of SMO. Active SMO promotes a signalling cascade that ultimately leads to translocation of full length (FL) activated forms of GLI (GLI^A^, green) into the nucleus, where they induce transcription of HH target genes. Abbreviations: CK1, casein kinase 1; GSK3β, glycogen synthase kinase 3β; HH, Hedgehog; PCAF, p300/CREB-binding protein (CBP)-associated factor; PKA, protein kinase A; PTCH, Patched; SMO, Smoothened; Spop, speckle-type POZ protein; SUFU, Suppressor of Fused; β-TrCP, β-transducin repeat-containing protein.

The initiation of the HH signalling begins with the binding of one of the three HH ligands, each with distinct spatial and temporal expression patterns, to the 12-pass transmembrane protein receptor PTCH, which resides in the primary cilium, a non-motile structure that functions as a sensor and coordinator centre for the HH signalling (Refs 9, 10, 11). Binding of HH ligands to PTCH relieves its inhibitory effect on the G-protein-coupled receptor-like SMO, which moves into the tip of the cilium and triggers a cascade of events that promote the formation of GLI activator forms (GLI-A). GLI2/3-A translocate into the nucleus and induce HH pathway target genes, including *GLI1* (Refs 12, 13, 14) (Fig. 1). In absence of HH ligands, PTCH inhibits pathway activation by preventing SMO to enter the cilium. This results in the phosphorylation and proteasome-mediated carboxyl cleavage of GLI3 and, to a lesser extent, of GLI2 to their repressor forms (GLI2/3-R; Refs 15, 16). GLI1 is degraded by the proteasome and is transcriptionally repressed, with consequent silencing of the pathway. GLI1 acts exclusively as an activator, whereas GLI2 and GLI3 display both positive and negative transcriptional functions (Refs 15, 17, 18) (Fig. 1).

The HH target genes include *GLI1*, which further amplifies the initial HH signalling at transcriptional level and, therefore, is a reliable and robust read-out of an active pathway (Ref. 19). Other HH target genes are *PTCH1* and HH interacting protein (*HHIP1*), which both mediate negative feedback by limiting the extent of HH signalling. The outcome of the HH signalling varies according to the receiving cell type, and includes a number of cell-specific targets mediating a variety of cellular responses: proliferation and differentiation (Cyclin D1 and D2, E2F1, N-Myc, FOXM1, PDGFRα, IGFBP3 and IGFBP6, Hes1, Neogenin), cell survival (BCL-2), self-renewal (Bmi1, Nanog, Sox2), angiogenesis (Vegf, Cyr61), cardiomyogenesis (MEF2C), epithelial–mesenchymal transition (Snail1, Sip1, Elk1 and Msx2) and invasiveness (Osteopontin). (Ref. 20). The strength of HH signalling is tuned by a number of post-transcriptional and post-translational modifications of the three GLI transcription factors.

### The GLI transcription factors and their modifications

The three GLI transcription factors are members of the Kruppel family. They share five conserved C_2_–H_2_ zinc-finger DNA-binding domains and a consensus histidine/cysteine linker sequence between the zinc fingers, and bind to the consensus motif GACCACCCA in the promoter of their target genes (Ref. 21). The sequence specificity of the GLI transcription factors, although, is not absolute, because they can recognise variant GLI-binding sites with relatively low affinity, still leading to strong transcriptional transactivation (Ref. 22).

GLI1 is a transcriptional target of GLI2 and GLI3 (Refs 17, 23) and a strong transcriptional activator. In both human and mouse cells, GLI1 protein is translated from alternative mRNAs that differ in their 5′ untranslated region and that are generated by exon skipping. The shorter mRNA shows the highest translation efficiency and it is the predominant transcript in proliferating cells and in basal cell carcinoma (BCC; Ref. 24). GLI1 mRNA also undergoes adenosine deamination acting on RNA (ADAR)-dependent A-to-I editing at position 2179. The consequent arginine-to-glycine amino acidic change at position 701 produces a GLI1 protein less sensitive to the inhibitory effect of Suppressor of Fused (SUFU) and with higher transcriptional activity (Ref. 25).

The subcellular localisation of GLI1 is tightly controlled. GLI1 nuclear localisation is observed upon HH stimulation and correlates with high transcriptional activity, whereas in absence of HH pathway activation GLI1 is retained in the cytoplasm and degraded by the proteasome. SUFU, one of the main negative regulators of HH signalling, interacts with GLI1 both at the N-terminal (amino acids 116–125) (Ref. 26) and at the C-terminal region (Refs 27, 28) and inhibits GLI1 both by retaining it in the cytoplasm and by repressing its transcriptional activity in the nucleus (Refs 29, 30).

GLI1 stability and proteasomal degradation is controlled by multiple factors. In absence of HH ligands, β-transducin repeat-containing protein (β-TrCP) E3 ubiquitin ligase recognises two sequences on GLI1 (degron N and C) and induces its proteasome-dependent degradation (Ref. 31). Likewise, NUMB targets GLI1 for proteasome degradation through the recruitment of the HECT-type E3 ubiquitin ligase ITCH (Ref. 32). Upon genotoxic stress, p53 induces the acetyltransferase p300/CREB-binding protein (CBP)-associated factor (PCAF), identified as a novel E3 ubiquitin ligase targeting GLI1 for proteasomal degradation (Ref. 33). At the same time, PCAF itself is required for the expression of HH target genes, because it acetylates histone H3K9 on promoters of HH targets (Ref. 34). Recently, the pro-apoptotic protein Fem1b has been found to interact with mammalian Gli1 and to promote its proteasomal degradation, leading to Hh signalling inhibition (Ref. 35).

GLI1 undergoes several post-translational modifications that modulate its activity. Deacetylation of GLI1 at Lys518 by histone deacetylase 1 (HDAC1) increases its transcriptional activity (Ref. 36). The serine/threonine unc-51-like kinase 3 (ULK3) enhances GLI1 (and GLI2) transcriptional activity and promotes GLI1 nuclear localisation (Ref. 37). Protein kinase A (PKA) phosphorylates GLI1 at Thr374, near the nuclear localisation signal, retaining it in the cytoplasm and inhibiting its transcriptional activity (Ref. 38). PKA also phosphorylates GLI1 on Ser640, and Gli2 and GLI3 on homologous sites, allowing their interaction with 14-3-3ε and leading to suppression of HH signalling (Ref. 39). Activation of GLI1 is observed upon phosphorylation on Ser243 and Thr304 by atypical protein kinase C (aPKC) ι/λ (Ref. 40) and on Ser84 by ribosomal protein S6 kinase 1 (S6K1) (Ref. 41). The dual specificity Yak-1 related kinases 1 (DYRK1) and 2 (DYRK2) modulate HH pathway in opposite ways. DYRK1 phosphorylates GLI1 in its N- and C-terminal regions, increasing its nuclear retention and transcriptional activity (Ref. 42), whereas DYRK2 reduces Gli1 transcriptional activity (Ref. 43).

GLI2 protein has the repressor domain at the N-terminus and the activator domain at the C- terminus. It acts as an activator or, in its C-terminal deleted form, as a repressor (Ref. 44). In absence of HH ligands, Gli2 is sequentially phosphorylated by PKA on a cluster of sites in its C-terminal domain. These modifications work as priming events for multiple adjacent casein kinase 1 (CK1) and glycogen synthase kinase 3β (GSK3β) phosphorylations (Refs 16, 45). The consequent Gli2 hyperphosphorylation allows the recruitment of β-TrCP, which targets Gli2 for proteasome-dependent cleavage generating the repressor form (Refs 16, 46). Gli2 is also phosphorylated by DYRK2 (Ser385 and Ser1011), which induces its degradation by the proteasome (Ref. 43). Stabilisation of GLI2 protein is observed upon activation of mitogen-activated protein/extracellular signal-regulated kinase 1 (MEK1)/extracellular signal-regulated kinase (ERK)/ribosomal S6 kinase 2 (RSK2) cascade. RSK2-mediated phosphorylation of GSK3β reduces its activity, leading to reduced GLI2 ubiquitination and processing, and to increased GLI2 nuclear localisation and activation (Ref. 47).

Gli2 subcellular localisation is controlled by SuFu, which binds to and retains Gli2 in the cytoplasm and inhibits its transcriptional activity in the nucleus (Ref. 30). SuFu also controls the protein stability of Gli2 by competing with speckle-type POZ protein (Spop), which binds to Gli2 and acts as an adaptor for Cul3-based E3 ubiquitin ligase, leading to Gli2 proteasomal degradation (Ref. 48). The interaction between SuFu and Gli2 is inhibited by Kif7, which acts as a positive regulator of Gli activity (Ref. 49). Kif7 itself, on the other hand, behaves as a negative regulator of Hh signalling, by binding to Gli2 and Gli3 and contributing to the efficient processing of Gli2 to its repressor forms (Ref. 50). GLI2 activity is also modulated by sumoylation and acetylation. PKA-dependent phosphorylation of Gli2 enhances conjugation of small ubiquitin-like modifier (SUMO) at Lys630 and Lys716. This results in the recruitment of HDAC5 with the consequent reduction of GLI2 transcriptional activity (Ref. 51). In absence of HH stimulation, p300 acetylates GLI2 at Lys757, reducing its chromatin recruitment and thus its transcriptional activity (Ref. 52).

GLI3 acts mostly as a repressor in its C-terminal cleaved form (Ref. 44). Nevertheless, in its full-length unprocessed form, it can mediate GLI1 induction upon Shh stimulation by interacting with the transcriptional activator CBP (Ref. 17). Gli3 processing is similar to that occurring to Gli2 and it is triggered by PKA-dependent phosphorylations, which are required for subsequent CK1 and GSK3β phosphorylations and recruitment of the β-TrCP ubiquitin ligase (Refs 15, 45, 53, 54). In this context, Kif7 plays a regulatory role in controlling the efficient relocalisation of Gli3 to the cilium in response to Shh and its processing to Gli3-R (Ref. 55). Like Gli1 and Gli2, Gli3 is also bound by Sufu (Ref. 29), which stabilises Gli3 and prevents Spop from promoting Gli3 degradation and processing to its repressor form (Ref. 48). The Gli3-dependent transcriptional repression involves the corepressor Ski, which interacts with Gli3 and likely recruits mSin3A, N-CoR/SMRT repressors leading to gene silencing (Ref. 56).

## Modes of action of HH-GLI signalling in cancer

Multiple mechanisms of HH pathway activation have been proposed in cancer (reviewed in Ref. 57). The mode of action of HH-GLI signalling has important implications for the design of therapeutic antagonists, therefore it is important to dissect the cellular and molecular mechanisms of HH activation in human cancers.

Ligand-independent activation (Type I) was the first type of aberrant HH pathway activation recognised in cancer, with the finding that patients with Gorlin syndrome (Ref. 58) harbour mutations in *PTCH1*. Tumours with ligand-independent activation of HH pathway carry genetic aberrations that confer cell-intrinsic growth properties to the tumour. The most frequent alterations found are inactivating mutations of pathway repressors, such as *PTCH1* (Refs 59, 60), *SUFU* (Refs 61, 62) or *REN* (Ref. 63), mutations leading to constitutive activation of *SMO* (Ref. 64), or gene amplifications of *GLI1* and *GLI2* (Refs 65, 66), that result in constitutive HH pathway activation. Defining the molecular mechanisms of ligand-independent activation of the signalling is crucial to determine whether a tumour might respond to the treatment with a HH inhibitor acting at the level of SMO or, in case the genetic alteration affects downstream components of the pathway, at the level of the GLI proteins.

Ligand-dependent autocrine/juxtacrine activation of the pathway (Type II) has been identified in the last few years in different types of cancers, including lung, pancreas, gastrointestinal tract, prostate and colon cancers, glioma and melanoma (Refs 62, 67, 68, 69, 70, 71, 72, 73, 74, 75, 76, 77, 78). In this case, tumours show increased HH ligand expression, in absence of genetic aberrations of HH pathway components, and respond to HH stimulation in cell-autonomous manner. This concept is supported by a number of experimental data showing that: (i) tumour cells, but not the surrounding stroma, express HH ligands and downstream HH signalling components (e.g. PTCH1, GLI1) (e.g. Refs 67, 74, 78); (ii) tumour cell growth could be inhibited by RNAi-mediated knockdown of SMO or GLI1 and by treatment with cyclopamine (a SMO antagonist) *in vitro* and in xenograft models *in vivo*; (iii) metastatic growth could be prevented *in vivo* upon RNAi-mediated knockdown of SMO or GLI1 (Ref. 67). These effects appear to be specific, because GLI1 epistatically rescues the inhibition of metastatic colonies obtained with SMO silencing (Ref. 67).

Ligand-dependent paracrine activation of HH pathway (Type IIIa) is a mode of action that resembles the physiological HH signalling occurring during embryo development. In this case, HH ligands secreted by cancer cells activate HH signalling in the surrounding stroma rather than in the tumour itself. The mechanisms by which the HH signalling pathway and the tumour stroma interact during paracrine signalling are not completely understood. However, activation of HH signalling in the tumour-associated stroma might lead to the production of growth factors (e.g. VEGF, IGF) and stimulation of other signalling pathways (e.g. Wnt, Interleukin-6) that in turn create a favourable microenvironment sustaining the growth and progression of the tumour (Ref. 79). Evidence supporting this mechanism has accumulated from studies in human tumour xenograft models of pancreatic and colorectal cancers that express high levels of HH ligands, in which increased expression of HH targets is detected specifically in tumour-infiltrating mouse stromal cells (Ref. 79). Interestingly, growth of mutant Kras-driven tumours is reduced in mice lacking Gli1 in the pancreatic microenvironment compared to wild-type mice (Ref. 80).

Similarly, the reverse paracrine HH pathway activation (Type IIIb) has been described in an experimental model of glioma (Ref. 81) and in haematological malignancies such as B-cell lymphoma and mantle cell lymphoma (MCL; Refs 82, 83). According to this modality, HH ligands are secreted by the tumour microenvironment (bone marrow stromal cells or endothelial cells) and activate the pathway on tumour cells, thus affecting its growth.

### HH-GLI signalling in cancer stem cells (CSCs)

Multiple lines of evidence indicate that HH-GLI pathway plays a role in the maintenance and regulation of CSCs in several types of cancer. Self-renewal, survival and tumourigenicity of CD133^+^ glioblastoma CSCs require SMO and GLI1 activity, as shown by their inhibition with cyclopamine and RNA interference (Refs 75, 84). Similarly, inhibition of SMO reduces epithelial–mesenchymal transition and self-renewal of glioblastoma-initiating cells (Ref. 85). CD44^+^/CD24^−/low^/Lin^−^ putative breast CSCs have higher levels of *GLI1* and *PTCH1* (Ref. 86). Pharmacological blockade of HH signalling with the SMO antagonist IPI-609 has shown a significant reduction in tumour engraftment rates of putative ALDH^high^ pancreatic CSCs (Ref. 87). Furthermore, CSCs with activated HH pathway have also been identified in multiple myeloma (MM; Ref. 88). Genetic studies in chronic myeloid leukaemia (CML) CSCs (Bcr-Abl-driven Lin^−^/Sca1^+^/c-Kit^+^) show that loss of SMO causes depletion of CML stem cells, whereas constitutively active SMO increases CML stem cell number and accelerates the disease (Refs 89, 90). Pharmacological inhibition of SMO reduces not only the propagation of CML driven by wild-type BCR-ABL, but also the growth of imatinib-resistant mouse and human CML (Ref. 90). Similarly, human B-cell acute lymphoblastic leukaemia (B-ALL) cell lines and clinical samples express HH pathway components and HH pathway blockade reduces B-ALL self-renewal *in vitro* and *in vivo* (Ref. 91). Clonogenic CD133^+^ colon CSCs express HH pathway components and require HH-GLI activity for their survival (Ref. 67). Both pharmacological inhibition of HH signalling with cyclopamine and GLI antagonist GANT61 and stable expression of RNAi targeting either SMO or GLI1 lead to a significant decrease of ALDH^high^ melanoma stem cell self-renewal and tumourigenicity (Ref. 92). Finally, inhibition of the HH-GLI pathway by cyclopamine reduces CD133^+^/CD15^+^ cell compartment and the tumourigenic capability of neuroblastoma cells (Ref. 93).

The critical tumourigenic role of HH pathway is further highlighted by its activity in CSCs, through the subverted regulation of stemness genes, such as NANOG and SOX2, which are overexpressed in certain cancer types. More specifically, the HH pathway has been shown to directly regulate *NANOG* transcription through GLI1 and GLI2 in neural stem cells (Ref. 94). In line with these findings, NANOG has been shown to act as a mediator of the HH-GLI signalling in regulating *in vivo* growth of glioblastoma CSCs (Ref. 95). Similarly, HH-GLI signalling regulates the expression of SOX2 in neural stem cells and medulloblastoma (Refs 96, 97). Recently, we showed that both GLI1 and GLI2 bind to *SOX2* promoter in melanoma cells and that SOX2 function is required for HH-induced self-renewal of melanoma CSCs (Ref. 98). Altogether, these findings suggest that aberrant HH signalling induces a number of stemness factors, that might play a critical role in the acquisition of a more undifferentiated and aggressive state through a process similar to reprogramming.

## Activation of HH-GLI signalling in human cancers

The initial link between HH signalling and cancer came from the finding that loss of function mutations in *PTCH1* gene are associated with a rare and hereditary form of BCC, basal cell nevus syndrome (BCNS) (also known as Gorlin syndrome) (Refs 59, 60, 99). BCNS is an autosomal dominant disorder with two distinct sets of phenotypes; increased risk of developing cancers such as BCC, medulloblastoma, rhabdomyosarcoma and meningioma, as well as developmental defects, including bifid ribs and ectopic calcifications (Ref. 58), that reflect the involvement of HH pathway in many developmental processes.

Consistent with the risk for specific cancers in Gorlin syndrome, sporadic BCCs and at least a subset of medulloblastomas (MBs), are the tumour types that show the strongest association with aberrant HH pathway activation, both in humans and in experimental mouse models. Activation of HH pathway in BCC and MB occurs through direct genetic alterations of HH pathway genes. Sporadic BCC and MB, a malignant brain tumour in children, harbour high frequency of inactivating mutations in *PTCH1* (Refs 99, 100, 101, 102, 103) or, to a lesser extent, activating mutations in *SMO* (Refs 64, 104), both leading to the constitutive activation of HH pathway. In addition, MBs also show mutations in *SUFU* (Ref. 61) and *GLI1* and *GLI2* amplifications (Ref. 105). Deletion of 17p region, which produces loss of the negative HH modulator REN(KCTD11), also leads to unrestrained HH signalling and uncontrolled proliferation of immature cerebellar granule neuron precursors cells (Ref. 106).

The genetic equivalent mouse model of BCNS, is a mouse heterozygous for a loss-of-function allele of *Ptch1*. Many of the BCNS features are recapitulated in this model, including occurrence of MB (Ref. 107), rhabdomyosarcoma (Ref. 108) and developmental aberrations. Notably, full-blown BCCs are rarely seen in *Ptch1^+/−^* mice maintained in normal conditions, but lesions resembling BCCs develop when mice are exposed to ultraviolet (UV) or ionising radiations (Ref. 109). This observation is in agreement with the clinical course of BCC in BCNS patients, where BCCs occur preferentially on sun-exposed areas of the body (Ref. 110). BCNS patients are predisposed to BCC, MB and rhabdomyosarcoma, but they are not at increased risk to develop other cancer types, such as glioma, breast or prostate cancers. Genetic mouse models and identification of genetic mutations in BCC and MB have suggested that aberrant activation of HH signalling is required and sufficient for the development of these cancers. In other types of cancer activation of HH signalling might require additional alterations/mutations in other signalling pathways to contribute to tumour development.

Glioma is the most frequent tumour of the central nervous system and can be classified into four grades, with glioblastoma multiforme (GBM) being the most aggressive. *GLI1* was originally identified as a gene amplified in malignant GBM (Ref. 65), although its amplification is detected in a small fraction of gliomas (Refs 111, 112). The landscape of driver genomic alterations in glioblastoma has been recently revealed, suggesting that ligand-independent activation of the HH pathway is not frequent (Ref. 113). Nevertheless, several reports support a role for HH signalling in gliomas. For instance, expression of components of HH signalling is observed in gliomas of different grades, with SHH expression mostly confined to the surrounding endothelial cells and astrocytes. Activation of the pathway sustains growth, survival and stemness of glioma cells and progenitors (Refs 75, 77, 84). Consistently, inhibition of HH signalling by cyclopamine treatment or by overexpression of miR-326, which targets SMO, decreases glioma growth, stemness and tumourigenicity (Refs 75, 84, 114).

There are strong indications that the HH pathway is involved also in human breast cancer (BC), the leading cause of cancer death among women. High expression of components of HH pathway, including GLI1, is associated with a higher risk of recurrence after surgery and poorer prognosis (Refs 115, 116, 117). Consistently, transgenic mice that conditionally express *GLI1* in the mammary epithelium develop mammary tumours (Ref. 118). Activation of HH signalling in BC results from genetic alterations, such as loss of *PTCH1* or *GLI1* amplification (Refs 119, 120) or from ligand-dependent stimulation. Indeed, invasive BC, but not normal breast epithelium, shows high expression of SHH, PTCH1 and GLI1 (Ref. 121). The elevated expression of HH ligands is associated with the development of a basal-like BC phenotype and to a poor prognosis (Ref. 115), and may result from hypomethylation of *SHH* promoter (Refs 122, 123) or from HH up-regulation mediated by transcription factors, such as nuclear factor kappa-light-chain-enhancer of activated B cells (NF-kB) (Ref. 123), p63 (Ref. 124) or Runx2 (Ref. 125). In addition, other cellular pathways contribute to directly activate the downstream effectors of the pathway. In oestrogen receptor (ER)-positive BC cells, oestrogen stimulation induces GLI1, which promotes CSC self-renewal and invasiveness (Ref. 126). In tamoxifen-resistant ER positive BC cells, ligand-independent activation of HH pathway results from phosphoinositide 3-kinase (PI3K)/AKT pathway (Ref. 127). Several reports point out the importance of paracrine HH signalling in BC, whose occurrence is associated to poor prognosis (Ref. 115). HH ligands are often expressed by the tumour epithelium, whereas the highest levels of SMO, GLI1 and GLI2 are found in the stroma (Ref. 128). GLI1 promotes vascularisation by inducing the pro-angiogenic factor CYR61 (cysteine-rich angiogenic inducer 61) (Ref. 129), which influences the tumour microenvironment. In addition, the alternatively spliced, truncated tGLI1 that is frequently expressed in BC, but not in normal tissue, induces the migration-associated genes VEGF-A and CD24 (Ref. 130).

Pancreatic ductal adenocarcinoma (PDAC) is an aggressive tumour that develops from pancreatic intraepithelial neoplasia (PanIN), characterised by frequent mutations of *KRAS, CDKN2A, TP53* and *SMAD4* (Ref. 131). A comprehensive genomic analysis revealed few missense mutations in *GLI1* and *GLI3*, whose oncogenic function remains to be determined (Ref. 132). Nevertheless, HH signalling is involved in pancreatic development (Ref. 133) and cooperates with oncogenic KRAS during the early stages of PDAC formation (Ref. 134). The ligands SHH and IHH are expressed in the duct epithelium of PanIN lesions and in PDAC, but not in normal human pancreas (Refs 70, 72). Human pancreatic cell lines produce SHH, IHH and show detectable level of the target genes *GLI1, PTCH1* and *HHIP*, indicating HH pathway activity. Moreover, proliferation and metastatic behaviour of some of these cell lines can be blocked by cyclopamine both *in vitro* and *in vivo* (Refs 70, 71, 72), supporting a ligand-dependent autocrine mode of action. Multiple evidence indicates the presence of paracrine HH signalling in pancreatic cancer. HH ligands produced by tumour cells activate HH pathway in the surrounding stroma, thus inducing the expression of HH targets that promote perineural invasion and metastasis (Refs 79, 135, 136, 137). Paracrine HH signalling also promotes the formation of desmoplasia, which contributes to the failure of the standard therapy (Ref. 138); indeed, chemical inhibition of HH pathway enhances the efficacy of chemotherapy (Ref. 139). However, recent data obtained in murine models of PDAC propose a controversial role for HH signalling in PDAC. In fact, activation of HH signalling has been shown to induce stromal hyperplasia and reduce epithelial growth, thus restraining tumour. Conversely, HH pathway inhibition accelerates tumour progression because, although reducing desmoplasia, it promotes proliferation and vascularisation of the tumoural epithelium, which exhibits a more undifferentiated phenotype (Refs 140, 141).

HH signalling is involved in prostate cancer (PC). Aberrant activation of HH signalling in PC might result from loss of *SUFU* or by ligand-dependent activation of the pathway due to high expression of SHH (Ref. 62). However, it is not clear whether HH activation occurs in a paracrine and/or autocrine/juxtacrine manner. Evidence suggests that the PC cells secrete HH ligands that activate the pathway in the surrounding stromal cells, which in turn produce factors promoting cancer cells proliferation (Refs 142, 143, 144). Conversely, other reports indicate the presence of a cell-autonomous activation of HH signalling in PC cells, whose proliferation is greatly decreased by cyclopamine treatment. The expression of HH ligands and of target genes in the tumour epithelium is higher than in the normal adjacent tissue and correlates with Gleason score, metastasis and poor prognosis (Refs 62, 73, 74, 145). The HH effector GLI2 is highly expressed in PC where it enhances proliferation, cell survival and tumourigenicity (Refs 146, 147). Multiple evidence suggests an interplay between HH and androgen signalling. Long-term androgen deprivation in PC leads to a strong up-regulation of HH signalling, which is also observed in androgen-independent (AI) PC cells (Refs 145, 148, 149). Overexpression of GLI1 and GLI2 enhances androgen-specific gene expression, indicating that HH signalling supports androgen signalling even in absence of androgen and in AI prostate cancer cells (Ref. 150).

HH pathway plays a role also in the most lethal form of skin cancer, malignant melanoma. A recent global genomic screening of 100 melanomas revealed few missense mutations in the core genes of the HH pathway (*PTCH1, SMO, SUFU, GLI1, GLI2* and *GLI3*) (Ref. 151), although their potential oncogenic function remains to be determined. Several studies report an active role for HH signalling in melanoma. Human melanomas express components of HH pathway (Ref. 78) and about half of melanoma cell lines express high levels of *SMO, GLI2* and *PTCH1* and low levels of the negative regulators *PKA* and *DYRK2* compared to melanocytes (Ref. 152). Interestingly, high HH pathway activity is associated with decreased post-recurrence survival in metastatic melanoma patients (Ref. 152). Moreover, we previously showed that growth and metastasis of human melanomas xenografts in nude mice can be blocked by local or systemic treatment with cyclopamine. Cyclopamine treatment drastically reduces tumour growth also in melanomas induced by oncogenic NRAS in a *Tyrosinase-NRAS^Q61K^; Ink4a^−/−^* mouse model (Ref. 78). Two recent studies confirmed and extended these findings; the SMO antagonist sonidegib has shown to reduce proliferation of human melanoma cell lines and to decrease human melanoma xenograft growth in nude mice (Refs 152, 153). Interestingly, one of the two studies showed a stronger inhibition of proliferation in BRAF mutant cell lines than in BRAF wild-type cells and a modest but significant effect combining BRAF and Hedgehog inhibitors (Ref. 152), suggesting that a combined therapy targeting both mutant BRAF and HH pathway could be beneficial in patients with mutated BRAF and activated HH signalling. Activation of HH pathway might also play a role in melanoma progression, by contributing to the acquisition of an invasive behaviour. Melanoma cells with high GLI2 expression are characterised by an invasive and metastatic phenotype, associated with loss of E-cadherin and secretion of metalloproteases, and metastasise to bone more quickly than cells with low GLI2 expression (Ref. 154). Furthermore, GLI1-mediated induction of Osteopontin correlates with tumour progression and metastasis of human melanomas (Ref. 155).

High expression of components of HH pathway is observed in colon cancer, where it correlates with poor prognosis and overall survival (Refs 156, 157). Autocrine HH signalling in colon cancer promotes cell growth, self-renewal of CSCs and metastatic behaviour (Ref. 67). Consistently, inhibition of GLI1 and GLI2 function induces apoptosis and DNA damage response in colon cancer cell lines (Ref. 158). Gastric cancers express high levels of PTCH1 and SHH (Refs 72, 159) and active HH signalling correlates with metastatic behaviour and poor prognosis (Refs 160, 161, 162, 163). Activation of the pathway results from *SMO* and *PTCH1* mutations (Ref. 164) or methylation of the promoter of the negative regulators *PTCH1* and *HHIP* (Refs 165, 166). Direct activation of GLI1 may also result from MAPK signalling, which leads to the induction of HH targets (Ref. 167), such as Bcl-2 (Ref. 168). HH signalling promotes gastric cancer cell growth and proliferation *in vitro* and *in vivo* (Ref. 169) and is highly active in gastric CSCs, where it is required for their self-renewal and resistance to chemotherapy (Refs 170, 171).

Lung cancer is the malignancy with the highest mortality and includes small-cell lung cancer (SCLC) and non-small-cell lung cancer (NSCLC). In a fraction of SCLC, ligand-dependent activation of HH signalling drives tumour growth *in vivo* and *in vitro* (Ref. 68). In NSCLC the expression of HH signalling components is higher than in the non-tumoural parenchyma and associates with high grade, poor survival and metastases (Refs 172, 173). In NSCLC, GLI1 regulates cell proliferation in cell-autonomous manner. Moreover, increased production of SHH by tumour cells leads to activation of fibroblasts in the tumour-associated stroma, indicating the presence of paracrine HH signalling (Refs 69, 174).

Aberrant activation of HH signalling has been observed in ovarian cancer (Refs 175, 176, 177) and high expression of its components correlates with poor clinical outcome (Refs 175, 178, 179). HH pathway has been shown to be involved in different aspects of ovarian carcinogenesis, by controlling proliferation and survival of ovarian carcinoma (Ref. 175), growth of cancer spheroid forming cells (Ref. 180), cell migration and invasion, through integrin β4-mediated activation of focal adhesion kinase (FAK; Ref. 181), and drug sensitivity, through regulation of the ATP-binding cassette transporter ABCB1 and ABCG2 (Ref. 182).

HH signalling is involved also in haematological malignancies. Increased HH activity has been reported in different haematological diseases, including CML (Ref. 183), acute myeloid leukaemia (AML) (Ref. 184), acute lymphocytic leukaemia (ALL) (Ref. 91), MM (Ref. 88), chronic lymphocytic leukaemia (CLL; Ref. 185), Hodgkin's lymphoma (Ref. 186), MCL (Ref. 83), diffuse large B-cell lymphoma (DLBCL) (Ref. 187) and ALK+ anaplastic large cell lymphoma (ALCL) (Ref. 188). The activation of HH signalling in these diseases likely results from the integration of deregulated oncogenic inputs that contribute to the direct activation of the GLI proteins. Different haematological malignancies also show different modalities of HH signalling activation, which has been proposed to be paracrine mainly in CLL and plasma cell myeloma, both paracrine and autocrine in DLBCL and autocrine in ALL, AML and ALK+ALCL.

## Modulation of HH-GLI signalling by oncogenic pathways

**Figure 2.**
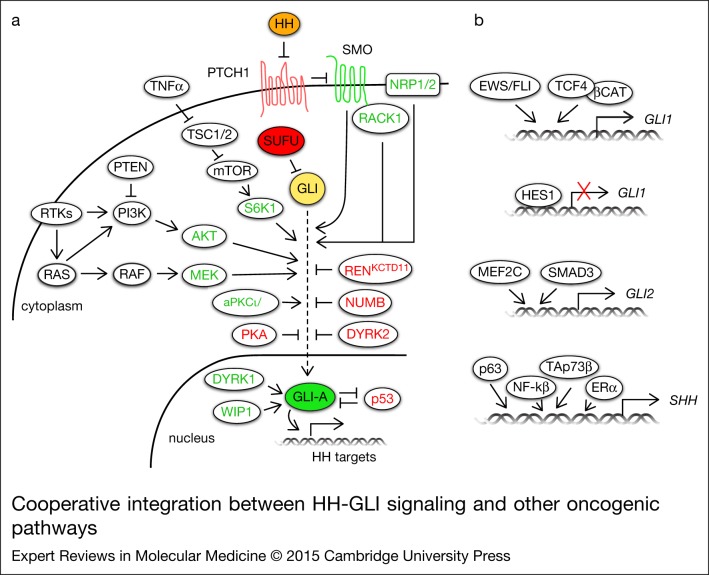
**Cooperative integration between HH-GLI signalling and other oncogenic pathways**. (a) Schematic diagram of the basic components of the HH-GLI signalling (filled circles) and their positive (in green) and negative regulators (in red) (unfilled circles). (b) Direct transcriptional regulators of *GLI1, GLI2* and *SHH*. See text for further details. Abbreviations: AKT, v-akt murine thymoma viral oncogene homologue; aPKCι/λ, atypical protein kinase C-ι/λ; β-CAT, β-catenin; DYRK1/2, dual specificity Yak-1 related kinase 1/2; ERα, oestrogen receptor α; EWS/FLI1, Ewing's sarcoma/friend leukaemia integration 1 transcription factor fusion gene; HES1, hairy and enhancer of split-1; HH, Hedgehog; mTOR, mammalian target of rapamycin; MEF2C, myocyte enhancer factor 2C; MEK, mitogen-activated protein/extracellular signal-regulated kinase; NF-kB, nuclear factor kappa-light-chain-enhancer of activated B cells; NRP1/2, neuropilin; PI3K, phosphoinositide-3-kinase; PKA, protein kinase A; PTCH, Patched; PTEN, phosphatase and tensin homologue; RACK1, receptor for activated C kinase 1; RTK, receptor tyrosine kinase; S6K1, ribosomal protein S6 kinase 1; SHH, Sonic hedgehog; SMO, Smoothened; SUFU, Suppressor of Fused; TNFα, tumour necrosis factor α; TSC1/2, tuberous sclerosis 1/2; WIP1, wild-type p53-induced phosphatase 1.

The activity of HH-GLI signalling observed in human cancer is the result of its functional interaction with other pathways and of the direct or indirect regulation of the final effectors of the HH signalling by oncogenes and tumour suppressors (Fig. 2). Multiple lines of evidence support an interplay between HH-GLI and PI3K/AKT or RAS/RAF/MEK signalling. PI3K/AKT negatively regulates the degradation of GLI2 by interfering with PKA/GSK3β-mediated phosphorylation of GLI2, which targets the protein to proteasome-mediated degradation (Ref. 189). In zebrafish, a constitutively active form of Akt1 synergises with activated Smo in tumour formation (Ref. 190). AKT1 potentiates GLI1 transcriptional activity and nuclear localisation in melanoma cells (Ref. 78). In contrast, GLI1 function is inhibited by PI3K/AKT2 signalling in neuroblastoma; AKT2 phosphorylates GSK3β and prevents the destabilisation of SUFU, resulting in reduced GLI1 nuclear localisation and transcriptional activity (Ref. 191).

An interplay between RAS/RAF/MEK and HH signalling has been described in various systems. For instance, oncogenic H- or N-Ras increase GLI1 function in melanoma cells and HH-GLI signalling is required for N-Ras-induced mouse melanoma growth (Ref. 78). Active K-Ras potentiates GLI1 activity in gastric cancer (Ref. 167) and in PDAC contributing to tumour progression (Ref. 192). K-Ras and activated HH signalling cooperate *in vivo* to initiate PDAC development (Ref. 193). An additional mouse model of K-Ras-induced PDAC shows that Smo-independent Gli1 activation is required for survival of tumour cells and K-Ras-mediated transformation. Interestingly, K-Ras and TGF-β were shown to regulate Gli1 expression in absence of Smo (Ref. 194). K-Ras also contributes to a shift from autocrine-to-paracrine signalling in PDAC: it induces SHH expression, thus leading to HH stimulation of adjacent cells, and negatively modulates canonical HH signalling through its effector DYRK1B (Ref. 195).

The ERK pathway positively modulates HH-GLI signalling. MEK1 increases GLI1 and GLI2 transcriptional activity (Ref. 196) and the ERK5 target myocyte enhancer factor 2C (MEF2C) directly regulates the expression of and cooperates with GLI2 during cardiomyogenesis *in vitro* (Ref. 197). Biochemical studies identified GLI1 and GLI3 as new MAPK substrates, because they can be phosphorylated *in vitro* by JNK1/2 and ERK2 (Ref. 198).

A crosstalk between epidermal growth factor receptor (EGFR) signalling and HH-GLI pathway has also been reported. In normal keratinocytes, EGFR signalling modulates HH-GLI target gene expression (Ref. 199) and during their transformation it induces activation of JUN/AP1, which cooperates with GLI1 and GLI2 (Ref. 200). Interestingly, a group of HH-EGFR cooperation response genes – SOX2, SOX9, JUN, CXCR4 and FGF19 – has been shown to determine the oncogenic phenotype of BCC and pancreatic CSCs (Ref. 201).

HH signalling is differently modulated by distinct members of the PKC family. Upregulation of aPKC ι/λ potentiates HH signalling by directly phosphorylating and activating GLI1. Because aPKC ι/λ is also an HH target gene, it sustains a positive feedback loop contributing to HH activation (Ref. 40). Similarly, PKCα increases GLI1 transcriptional activity in a MEK/ERK-dependent manner (Ref. 202). Conversely, PKCδ reduces GLI1 nuclear localisation and transcriptional activity, leading to suppression of HH signalling (Ref. 202).

Receptor for activated C kinase 1 (RACK1) interacts with and activates SMO, enhancing GLI1 function and increasing cell proliferation and survival in NSCLC (Ref. 203). A positive feedback regulation fuelling Hh signalling activation involves Neuropilin1 (Nrp1) and 2 (Nrp2). Activated Hh pathway induces Nrp1 and Nrp2, which in turn potentiate Hh signalling transduction acting between Smo and SuFu (Ref. 204). Activation of tumour necrosis factor alpha  (TNF-α)/mammalian target of rapamycin (mTOR) pathway in oesophageal carcinoma activates HH-GLI signalling through phosphorylation of GLI1 by S6K1, which induces its release from SUFU (Ref. 41).

Activation of HH-GLI signalling due to direct induction of GLI1 expression is observed after activation of WNT/β-catenin signalling (Ref. 205) and in Ewing Sarcoma Family Tumours (ESFT), where the oncogenic transcription factor EWS/FLI1, resulting from the chromosomal translocation t(11;22), directly induces GLI1 expression (Refs 206, 207) (Fig. 2b). Likewise, transforming growth factor β (TGF-β) stimulation leads to a SMAD3-dependent induction of GLI2, which in turn increases GLI1 expression (Ref. 208). TGF-β also induces Kindlin-2, which increases GLI1 protein levels by inhibiting GSK3β. GLI1, in turn, represses Kindlin-2 creating a regulatory loop (Ref. 209) (Fig. 2b). Activation of HH pathway in some tumours results from the increase of HH ligands. For instance, ERα pathway in gastric cancer (Ref. 210) or NF-kB in pancreatic cancer cells (Refs 211, 212) directly increase SHH expression, leading to enhanced proliferation and resistance to apoptosis. Direct induction of SHH is also mediated by p63β, p63γ and TAp73β, which bind to SHH promoter (Ref. 124) (Fig. 2b).

Although HH signalling activation is regulated by many phosphorylation events, only few phosphatases have been described to modulate the pathway. In *Drosophila* PP4 and PP2A act as negative and positive modulators of HH signalling, acting at the level of Smo and Ci, respectively (Ref. 213). Recently, the oncogenic wild-type p53-induced phosphatase 1 (WIP1) has been described to cooperate with SHH to enhance tumour formation in SHH-dependent medulloblastoma (Ref. 214). Our group showed that WIP1 phosphatase activity enhances GLI1 function in melanoma by increasing GLI1 nuclear localisation, protein stability and transcriptional activity, whereas its inhibition reduces self-renewal and tumourigenicity of melanoma cells with activated HH signalling (Ref. 215).

A negative reciprocal regulation is observed between GLI1 and the tumour suppressor p53. p53 inhibits the activity, nuclear localisation and protein levels of GLI1 in neural stem cells and glioblastoma cells (Ref. 216). Conversely, HH signalling inhibits p53 by inducing activating phosphorylations on MDM2, thus enhancing p53 degradation (Ref. 217). Inhibition of HH signalling results from activation of NOTCH pathway observed in glioblastoma and melanoma. The NOTCH target hairy and enhancer of split-1 (HES1) binds to the first intron of *GLI1*, repressing its expression (Ref. 218) (Fig. 2b). In BC, high levels of liver kinase B1 (LKB1) are associated with low levels of HH signalling activation (Ref. 219). Another suppressor of HH signalling is REN^KCTD11^, which is often deleted in medulloblastoma and it has been shown to retain GLI1 in the cytoplasm, reducing its transcriptional activity (Ref. 63).

Regulation of HH signalling occurs also at epigenetic level. Menin, the gene mutated in multiple endocrine neoplasia type 1, recruits the protein arginine methyltransferase 5 (PRMT5) to growth arrest-specific 1 (Gas1) promoter. The consequent Gas1 repression prevents the binding of Shh to Ptch1, thus resulting in reduced HH pathway activity (Ref. 220). Different components of HH signalling are also targets of micro-RNAs (miR). miR-125b and miR-326, which target SMO, and miR-324-5p, which targets both SMO and GLI1, are downregulated in HH-driven MB and contribute to sustain tumour growth (Ref. 221). In glioblastoma the miR-302-367 cluster inhibits clonogenicity and stemness of glioblastoma stem cells, through downregulation of CXCR4/SDF1 and consequent reduction of SHH, GLI1 and NANOG levels (Ref. 222).

## Inhibitors of HH-GLI signalling

**Figure 3.**
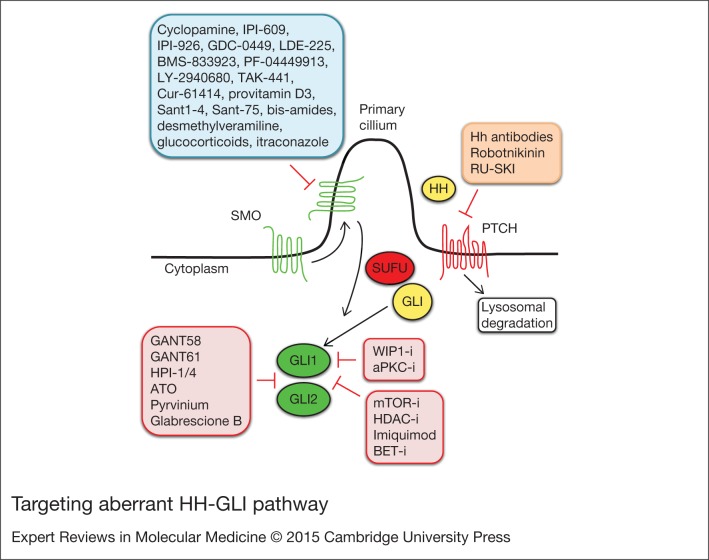
**Targeting aberrant HH-GLI pathway**. HH-GLI antagonists, classified according to what level of the pathway they inhibit: SMO translocation and activation (blue); HH/PTCH interaction (orange); GLI nuclear translocation and transcriptional activity (red). Abbreviations: aPKC-i, atypical protein kinase C-inhibitor; ATO, arsenic trioxide; BET-i, BET bromodomain inhibitor; HDAC-i, histone deacetylase-inhibitors; HH, hedgehog; HPI-1/4, hedgehog pathway inhibitors 1–4; mTOR-i, mammalian target of rapamycin inhibitors; PTCH, Patched; SMO, Smoothened; SUFU, Suppressor of Fused; WIP1-i, wild-type p53-induced phosphatase 1-inhibitors. See the main text for details.

Current HH pathway antagonists can be classified according to what level of the pathway they modulate: (i) HH/PTCH interaction; (ii) SMO translocation and activation; (iii) GLI nuclear translocation and transcriptional activation (Fig. 3).

### Acting at the level of SMO

The development of strategies targeting the HH signalling pathway began with the discovery that cyclopamine, a steroidal alkaloid derived from *Veratrum californicum* with teratogenic properties (Ref. 223), inhibits SMO (Refs 224, 225). Cyclopamine has been extensively used to study HH signalling and found to inhibit tumour growth in multiple *in vitro* and *in vivo* models. For instance, oral cyclopamine can block the growth of UV-induced BCCs in *Ptch^+/−^* mice by 50%, as well as inhibit the formation of new tumours (Ref. 226). Cyclopamine also reduces medulloblastoma development in *Ptch^+/−^* mice (Ref. 227) and decreases growth of many human cancer cell lines in xenotransplantation (Refs 70, 73, 75, 78, 228). However, cyclopamine is not suitable for clinical development because of its poor oral solubility. Efforts to improve the specificity, potency, and pharmacologic profile of cyclopamine have led to the synthesis of novel derivatives such as KAAD-cyclopamine (Ref. 229), IPI-609 (Ref. 87) and saridegib (IPI-926) (Ref. 230).

Additional SMO inhibitors are currently available and many, including vismodegib (GDC-0449), sonidegib (LDE-225), BMS-833923, PF-04449913 and LY2940680 are being investigated in clinical trials in a number of advanced cancers (Ref. 231) (Table 1). Among these, vismodegib is the first Hedgehog signalling antagonist approved by U.S. Food and Drug Administration (FDA) for treatment of advanced or metastatic BCC. Two SMO inhibitors, saridegib and TAK-441, have been discontinued for lack of efficacy (Refs 232, 233). A number of additional SMO antagonists have been used in preclinical studies; they include Cur-61414 (HhAntag; Refs 234, 235), provitamin D3 (Ref. 236), Sant1-4 (Ref. 237), Sant-75 (Ref. 238), bis-amide compound 5 (Ref. 239) and desmethylveramiline (Ref. 240). Glucocorticoids have recently been proposed as modifiers of HH signalling and SMO ciliary translocation; one class promotes ciliary accumulation resulting in enhanced Hh ligands response, whereas a second class inhibits SMO ciliary accumulation and is active against oncogenic and resistant SMO mutations (Ref. 241). Similarly, itraconazole, a common antifungal agent, has been identified as a potent inhibitor of the HH pathway by preventing ciliary translocation of SMO (Ref. 242). Systemic administration of itraconazole inhibits the growth of HH-dependent MB and BCC in mice and it is also active against drug-resistant mutant SMO D473H and Gli2 overexpression (Ref. 243).

**Table 1.** Selected clinical trials of SMO inhibitors in cancer

SMO inhibitor	Phase	Indication	Status^**a**^	Adverse events	Dose/efficacy	NCT # identifier^**b**^
Vismodegib (GDC-0449)	1	Locally advanced or metastatic solid tumours (*n *= 68)	Completed	Frequent grade 1/2 AEs: muscle spasms, dysgeusia, fatigue, alopecia, nausea, decreased appetite, diarrhoea; grade 3/4 AEs occurred in 37% of patients	Recommended phase 2 dose: 150 mg/day; ORR: 29% (58% in BCC, 1 unconfirmed response in MB) (Ref. 246)	NCT00607724
	1	Recurrent or refractory childhood MB	Completed		Recommended dose: 150–300 mg (Ref. 251)	NCT00822458
	1	High-risk first remission or relapsed multiple myeloma	Ongoing			NCT01330173
	2	Recurrent of refractory adult MB	Ongoing			NCT00939484
	2	Locally advanced or metastatic BCC (*n *= 96)	Ongoing	Frequent grade 1/2 AEs (>30% of patients): muscle spasms, alopecia, dysgeusia, weight loss, fatigue; grade 3/4 AEs were reported in 25% patients	Response rate: 43% for locally advanced BCC; 30% for metastatic BCC (Ref. 247)	NCT00833417
	2	Ovarian cancer in second or third remission (*n *= 104)	Completed	Common grade 1/2 AEs: dysgeusia, ageusia, muscle spasms, alopecia; grade 3/4 AEs occurred in 23% (vismodegib) and 11.5% (placebo) of patients	Median PFS: vismodegib 7.5 months compared with placebo 5.8 months (Ref. 249)	NCT00739661
	2	BCNS (*n *= 41)	Ongoing	Frequent AEs: dysgeusia, muscle cramps, alopecia, weight loss	Per patient rate of newly formed BCCs: 2 in vismodegib, 29 in placebo (Ref. 244)	NCT00957229
	2	Advanced chondrosarcoma	Ongoing	Common grade 1/2 AEs: myalgia, dysgeusia and alopecia; 2 patients had reversible grade 3/4 ALT or AST increase	Clinical benefit rate: 26%; median PFS: 3.5 months; median OS: 12.4 months (Ref. 250)	NCT01267955
Sonidegib (LDE-225)	1	Advanced solid tumours, including MB and BCC (*n *= 103)	Completed	Common grade 1/2 AEs: muscle spasms, myalgia, gastrointestinal toxicities, increased liver enzymes, dysgeusia, fatigue, alopecia	MTD: 800 mg/day; DLT: reversible grade 3–4 elevated serum creatine kinase (Ref. 248)	NCT00880308
	1/2	Advanced solid tumours in children, including MB	Ongoing			NCT01125800
	2	Relapsed/refractory acute leukaemia	Ongoing			NCT01826214
	2	ER/HER2-breast cancer	Recruiting			NCT01757327
BMS-833923	1	Solid tumours	Completed			NCT01413906
	1	Advanced or metastatic solid tumours, including BCC and BCNS	Completed			NCT00670189
PF-04449913	1	Advanced solid tumours (*n *= 23)	Completed	Frequent grade 1/2 AEs: dysgeusia, fatigue, decrease appetite, nausea, dizziness, dehydration, diarrhoea	Recommended dose: 80–320 mg/day (Ref. 300)	NCT01286467
	1	Haematologic malignancies	Completed			NCT00953758
LY2940680	1	Advanced solid tumours	Ongoing			NCT01226485

Abbreviations: AEs, adverse events; ALT, alanine transaminase; AST, aspartate transaminase; BCC, basal cell carcinoma; BCNS, basal cell nevus syndrome; DLT, dose-limiting toxicity; ER, oestrogen receptor; HER2, human epidermal growth factor receptor 2; MB, medulloblastoma; MTD, maximum tolerated dose; ORR, overall response rate; OS, overall survival; PFS, progression free survival.

^a^Status assessed on December 10th 2014; all ongoing trials are not recruiting.

^b^NCT # Identifier at http://clinicaltrials.gov.

#### SMO inhibitors in clinical development

SMO inhibitors are being investigated in clinical trials in a range of advanced cancers (Table 1). Several of these agents have induced tumour response in patients with tumours that harbour mutations in *SMO* and *PTCH1*, such as BCC and MB. Vismodegib drastically reduces the rate of appearance of new BCCs in patients with BCNS, without signs of resistance during treatment, in contrast with HH-dependent MBs. However, most BCCs have been shown to regrow after the drug is stopped (Ref. 244). In sporadic cases, 58% of patients with late advanced or metastatic BCC showed tumour regression in phase I clinical trials (Refs 245, 246) and 30% of metastatic and 43% of locally advanced BCC responded in phase II clinical trials (Ref. 247). These results suggest that tumours with low mutation rate such as in BCNS patients are predicted to respond well to SMO inhibition, whereas metastatic BCCs with high mutation rate have a higher likelihood to develop acquired resistance during treatment. Similar responses have been observed in BCC with sonidegib (Ref. 248). A phase II study evaluated vismodegib after chemotherapy in patients with ovarian cancer in second or third remission. However, the trial did not meet the primary endpoint and only a modest improvement in progression free survival was observed for vismodegib compared to placebo (7.5 versus 5.8 months). In addition, more than half of the patients discontinued treatment for disease progression and adverse effects (Ref. 249). Similarly, a phase II study of vismodegib in patients with advanced chondrosarcoma did not meet the primary endpoint (Ref. 250).

Tumour responses in MB have been reported with vismodegib and sonidegib (Refs 248, 251). Sonidegib has shown anti-tumoural activity in relapsed MBs associated with activated HH pathway, with dose- and exposure-dependent inhibition of *GLI1* expression (Ref. 248). A recent study showed the usefulness of a five-gene HH signature in formalin-fixed, paraffin-embedded tumour samples as a preselection tool for HH inhibitor therapy in MB patients (Ref. 252).

The use of SMO inhibitors has been associated with the acquisition of resistance to SMO inhibitors, mostly described in medulloblastoma, as a consequence of (i) mutations in human *SMO* (D473H) and the matching mutation in mouse (D477G), observed during vismodegib treatment (Ref. 253); (ii) amplification of downstream HH target genes, such as *GLI2* and *CyclinD1* (Refs 239, 254), reported for both vismodegib and sonidegib; (iii) upregulation of other oncogenic signalling, such as PI3K/AKT pathway (Ref. 254), observed during LDE-225 treatment; (iv) increased expression of adenosine triphosphate (ATP)-binding cassette transporter (ABC) such as P-glycoprotein, leading to increased drug efflux (Ref. 255), observed during saridegib treatment.

In studies investigating systemic treatments with SMO inhibitors, a common set of adverse effects has been observed, including muscle spasms, loss of taste (dysgeusia), hair loss (alopecia), fatigue, nausea, diarrhoea, decreased appetite, weight loss and hyponatraemia (summarised in Table 1). It is likely that hair loss, altered taste and diarrhoea are directly related to the inhibition of the intended molecular target (SMO), since HH signalling is known to be active in hair follicle, taste buds and gastrointestinal tract (Refs 256, 257, 258). Therefore, these effects are unlikely to be avoided by modifying the molecular structure of the agents. Possible strategies to lessen these effects would be to perform interval dosing of single agent or lower doses in combination with other agents (see later). Although most of the side effects of SMO inhibitors are mild to moderate (grade 1/2, Table 1), in some cases their severity has caused 50% of dropouts (Ref. 244) and raised concerns about long-term treatment in patients with BCC, typically a non-life-threatening cancer. One way to avoid or reduce such effects in BCC might be to use these inhibitors topically, limiting systemic exposure. A study employing topical treatment of LDE-225 for 4 weeks documented an effective reduction in tumour size or clinical clearing that correlated with effective inhibition of HH signalling (Ref. 259).

### Acting at the level of HH/PTCH interaction

Interference with the interaction between HH ligands and PTCH has been shown to attenuate HH signalling in experimental models. The monoclonal antibody 5E1 blocks the binding of HH ligands to PTCH1 with low nanomolar potency (Ref. 260). This antibody has been widely used in experimental studies to demonstrate HH dependency in tumour models, but it has not advanced to clinical settings. Recently, a novel neutralising antibody acting on SHH and IHH with low picomolar affinity has been reported (Ref. 261). Moreover, two small molecules have been described; robotnikinin binds to and inhibits SHH protein (Ref. 262), whereas RU-SKI, an inhibitor of HH acyltransferase, hampers SHH palmitoylation and blocks HH signalling (Ref. 263).

### Acting at the level of GLI

The development of molecules able to target directly the GLI, the final effectors of the HH signalling, would provide a good approach to block both canonical and non-canonical HH pathway activation and perhaps overcome anti-SMO drug resistance. Unfortunately, so far only few molecules acting on GLI proteins have been identified and their use is only limited to preclinical studies. A cell-based screening for inhibitors of GLI1-mediated transcription identified two structurally different compounds, GANT61 and GANT58. Both are capable of interfering with GLI1 and GLI2-mediated transcription and inhibit tumour cell growth in a GLI-dependent manner (Ref. 264). A screening of natural products identified physalins F and B as inhibitors of GLI-mediated transcriptional activity (Ref. 265). More recently, HPI-1/4 were described to act at or downstream of SUFU through various mechanisms, such as interfering with GLI processing or GLI activation. In particular, HPI-1 and HPI-4 have been shown to increase the proteolytic cleavage of Gli2 to its repressor form, whereas HPI-4 also decreases Gli1 stability (Ref. 266).

Arsenic trioxide (ATO), an already approved therapeutic for acute promyelocytic leukaemia, inhibits the GLI transcription factors (Refs 267, 268). Mechanistically, ATO directly binds to GLI1 protein and inhibits its transcriptional activity (Ref. 268) and blocks HH-induced ciliary accumulation of GLI2 (Ref. 267). The *in vivo* efficacy of ATO was demonstrated in both studies; it inhibits the growth of Ptch^+/−^/p53^−/−^ medulloblastoma allografts and Ewing sarcoma xenografts and increases survival of constitutively activated SMO transgenic mice with MB (Refs 267, 268).

Pyrvinium, an FDA-approved anti-pinworm agent, has recently been shown to inhibit Gli activity and enhance Gli degradation in a CK1α-dependent manner (Ref. 269). Consistent with its activity on the downstream mediators of the HH signalling, pyrvinium is able to inhibit the activity of a vismodegib-resistant SMO mutant (D473H) and Gli activity resulting from loss of Sufu, as well as to reduce *in vivo* growth of *Ptch^+/−^* MB allografts (Ref. 269).

Recently, the structural requirements of Gli1 for binding to DNA where clarified and a small molecule (Glabrescione B) that binds Gli1 zinc finger and interferes with its interaction with DNA was identified (Ref. 270). Glabrescione B is an isoflavone naturally present in the seeds of *Derris glabrescens*. Remarkably, as consequence of its strong inhibition of Gli1 activity, Glabrescione B inhibits growth of Hh-dependent BCC and MB tumour cells *in vitro* and *in vivo* as well as self-renewal ability and clonogenicity of CSCs (Ref. 270).

Inhibition of BET bromodomain proteins has recently emerged as a novel strategy to target epigenetically the Hh pathway transcriptional output (Ref. 271). The BET bromodomain protein BRD4 is a critical regulator of *GLI1* and *GLI2* transcription through direct occupancy of their promoter. Interestingly, occupancy of *GLI1* and *GLI2* promoters by BRD4 and transcriptional activation at cancer-specific GLI promoter-binding sites are markedly inhibited by the BET inhibitor JQ1. In Ptch-deficient MB and BCC mouse models and patient-derived tumours with constitutive HH pathway activation, JQ1 decreases tumour cell proliferation and viability *in vitro* and *in vivo*, even in presence of genetic alterations conferring resistance to SMO inhibition (Ref. 271). These findings suggest that BET inhibition could be effective against tumour cells that evade SMO antagonists through mutation of *SMO* or amplification of *GLI2* and *MYCN*, although the potential toxicities of BET inhibitors remain to be elucidated.

### Acting on other proteins/pathways that modulate HH signalling

Other compounds might inhibit HH signalling by targeting proteins and/or pathways that modulate GLI transcription factors. For instance, forskolin inhibits HH signalling by activating PKA, which in turn is involved in the phosphorylation of GLI2/GLI3, leading to their proteolytic processing into C-terminally truncated repressor forms (Ref. 272). Similarly, imiquimod, a nucleoside analogue of the imidazoquinoline family approved for treatment of BCC (Ref. 273), has been shown to induce a PKA-mediated GLI phosphorylation with consequent reduction in GLI activator levels (Ref. 274). Myristoylated aPKC peptide inhibitor (PSI) inhibits phosphorylation and activation of GLI1 by aPKC-ι/λ in BCC (Ref. 40). Rapamycin inhibits TNF-α-induced and mTOR-S6K1, mediated phosphorylation and activation of GLI1 in oesophageal adenocarcinoma (EAC) cells (Ref. 41).

## Evidence for rational combinations

### Combination of SMO inhibitors and other agents in preclinical studies

Support for combinatorial strategies is derived from the increasing amount of experimental data showing evidence of non-canonical HH signalling activation in tumours (summarised in Table 2). Combined inhibition of HH and MEK or AKT has been shown to yield additive/synergistic effects in reducing melanoma and cholangiocarcinoma cell proliferation *in vitro* (Refs 78, 275). Combination of EGFR and SMO inhibitors has been described in several preclinical models. In pancreatic cancer cells, treatment with cyclopamine and EGFR inhibitor gefinitib decreased tumour growth rate and increased apoptosis (Ref. 276). Treatment of prostate cancer cells with cyclopamine in combination with gefitinib and docetaxel cooperatively inhibited proliferation and invasiveness (Ref. 277). Combination of cyclopamine with erlotinib or sequential treatment with erlotinib followed by cyclopamine inhibited tumour-initiating potential in glioblastoma cells (Ref. 278). Similarly, combination of the SMO inhibitor saridegib and EGFR inhibitor cetuximab drastically decreased head and neck squamous cell carcinoma tumour growth *in vivo* (Ref. 279).

**Table 2.** Examples of preclinical combination studies of SMO inhibitors and other agents

Inhibitors (target)	Indication	Effects in preclinical study	References
*HHi + RAS/RAF/MEKi*			
Cyclopamine + U0126 (MEK)/SH6 (AKT)	Melanoma	Combination synergistically reduces cell growth *in vitro*	(78)
Cyclopamine + U0126 (MEK)	Cholangiocarcinoma	Additive antiproliferative effect	(275)
*HHi + EGFRi*			
Cyclopamine + Gefitinib	Pancreatic cancer	Combination decreases cell viability *in vitro* and tumour growth *in vivo*	(276)
Cyclopamine + Gefitinib + Docetaxel	Prostate cancer	Combination decreases tumour growth and invasion *in vitro*	(277)
Cyclopamine + Erlotinib	GBM	Combination decreases tumour sphere formation	(278)
Saridegib + Cetuximab	HNSCC	Combination abrogates tumour growth *in vivo*	(279)
*HHi + PI3K/mTORi*			
Sonidegib + Buparlisib (PI3K) or BEZ235 (PI3K/mTOR)	MB	Combination delays resistance observed with sonidegib alone	(254)
Sonidegib + Buparlisib (PI3K)	GBM	Combination synergistically reduces tumour growth *in vitro* and *in vivo*	(280)
Cyclopamine or CUR199691 + Rapamycin (mTOR) + Gemcitabine	Pancreatic cancer	Triple combination leads to CSC elimination *in vitro* and *in vivo* and increases survival	(281)
Vismodegib + Everolimus (mTOR)	Oesophageal adenocarcinoma	Everolimus enhances vismodegib inhibition *in vivo*	(41)
SIBI-C1 + Rapamycin (mTOR) + Gemcitabine	Pancreatic cancer	Triple combination eliminates CSC, reduces tumour growth *in vivo* and increases survival	(282)
*HHi + NOTCHi*			
Cyclopamine + GSI-XXI	Leukaemia	Combination inhibits growth *in vitro*	(283)
Cyclopamine + MRK-003	GBM	Combination decreases cell growth and increases apoptosis	(218)
Cyclopamine + GSI-1	Glioma	Combination enhances the effect of temozolomide	(284)
Cyclopamine or Vismodegib + DBZ or compound E	Prostate	Combination depletes docetaxel-resistant prostate cancer cells *in vitro* and *in vivo*	(285)
*HHi + BCR-ABLi*			
Cyclopamine + Nilotinib	CML	Combination prolongs survival in mouse model	(89)
Vismodegib + Dasadegib	CML	Combination enhances cytotoxicity	(288)
Vismodegib + Ponatinib (pan-ABL)	Leukaemia	Combination leads to decreased CD19+ cells, overall tumour burden and increases survival	(289)
*HHi + others*			
Cyclopamine + Gemcitabine	Pancreatic cancer	Combination abrogates metastases and reduces primary tumours	(71)
Saridegib + Gemcitabine	Pancreatic cancer	Saridegib facilitates gemcitabine delivery and extends survival	(139)
Cyclopamine + Temozolomide	GBM	Combination synergistically reduces CSC proliferation *in vitro*	(75)
SEN-450 + Temozolomide	GBM	Combination reduces tumour growth *in vivo*	(290)
Sonidegib + VX-680 (Aurora kinase) or BI-2536 (Polo-like kinase)	MB	Combination reduces proliferation *in vitro* and *in vivo*	(292)
Cyclopamine + CCT (WIP1)	Melanoma, breast cancer	Combination synergistically reduces tumour growth *in vitro*	(215)

Abbreviations: CCT, CCT007093; CML: chronic myeloid leukaemia; CSC, cancer stem cells; GBM: glioblastoma; HNSCC, head and neck squamous cell cancer; MB, medulloblastoma.

Several preclinical studies have evaluated HH pathway inhibitors in combination with PI3K and mTOR inhibitors. Simultaneous treatment with sonidegib and the PI3K inhibitor buparsilib (BKM120) or the dual mTOR/PI3K inhibitor BEZ235 led to a significant delay in resistance development (Ref. 254). Similarly, HH and PI3K pathways have been shown to synergise in promoting tumour growth in PTEN-deficient glioblastomas, and combined inhibition of the two pathways resulted in improved efficacy compared with inhibition of either pathway alone (Ref. 280). In pancreatic cancer, combination of chemotherapy, cyclopamine and the mTOR inhibitor rapamycin led to a near complete elimination of CSCs and increased long-term survival in mouse model (Ref. 281). Combination of vismodegib and the mTOR inhibitor everolimus resulted into a better response than each treatment alone in EAC xenografts (Ref. 41). Recently, multimodal treatment with the novel HH pathway inhibitor SIBI-C1, the mTOR inhibitor rapamycin and gemcitabine was shown to eliminate pancreatic CSCs and to increase survival of primary human pancreatic cancer tissue xenografts (Ref. 282).

Combination of HH and Notch inhibitors has also proved potential therapeutic efficacy in preclinical studies. For instance, cyclopamine and a γ-secretase inhibitor showed additive growth suppression in leukaemia cell lines (Ref. 283). Combined inhibition of cyclopamine and the γ-secretase inhibitor MRK-003 led to decreased glioblastoma cell growth, increased apoptosis and decreased colony formation compared with either agent alone (Ref. 218). Similarly, treatment of CD133+ glioblastoma stem cells with cyclopamine and a γ-secretase inhibitor enhanced the therapeutic effect of temozolomide (Ref. 284). Inhibition of Notch and Hedgehog signalling were also shown to affect docetaxel-resistant hormone-refractory prostate cancer cells, which have a high tumour-initiating potential. Treatment with the γ-secretase inhibitor DBZ or compound E and with cyclopamine or vismodegib reduced growth of docetaxel-resistant hormone-refractory prostate cancer cells *in vitro* and *in vivo* through inhibition of the survival molecules AKT and Bcl-2 (Ref. 285).

BCR-ABL tyrosinase kinase inhibitors (TKI) are effective against CML; however, these agents are unable to eliminate quiescent leukaemia stem cells (Ref. 286). Therefore, combination therapies with HH inhibitors are being explored. First of all, it was shown that imatinib-sensitive and -resistant CML cell lines express components of HH signalling, and genetic silencing of GLI1 reduced BCR-ABL protein expression, effect that is reversed by SMO agonist treatment (Ref. 287). Cyclopamine enhanced the effect of the BCR-ABL inhibitor nilotinib and prolonged the survival of mice by acting on leukaemic stem cells in a mouse model of CML (Ref. 89). SMO inhibition impaired propagation not only of wild-type BCR-ABL, but also of imatinib-resistant mouse and human CML (Ref. 90). In the BCR-ABL-positive cell line OM9;22, the combination of vismodegib with the BCR-ABL TKI dasatinib resulted in enhanced cytotoxicity compared with each drug alone (Ref. 288). Similarly, simultaneous treatment with vismodegib and the pan-ABL kinase inhibitor ponatinib reduced the percentage of CD19-positive leukaemia cells and overall tumour burden, and increased survival compared with treatment with either compound alone (Ref. 289).

Another example of combination drug for SMO inhibitors is gemcitabine for the treatment of pancreatic cancer. The activity of SMO inhibitor as a single agent in a pancreatic cancer xenograft model is modest and seems to be mediated by stromal pathway inhibition (Ref. 79). The combination of cyclopamine with gemcitabine completely abrogated metastases and significantly reduced the size of primary tumours in an orthotopic model of pancreatic cancer (Ref. 71). In a mouse model of pancreatic cancer (Trp53^R172H^ and Kras^G12D^) saridegib (IPI-926) was proposed to sensitise tumours to gemcitabine treatment through depletion of the tumour stroma (Ref. 139).

In light of the role of HH signalling in the maintenance of CSCs, combinatorial therapy with SMO antagonists and debulking chemotherapeutic agents has attracted interest, particularly with the respect to preventing relapse or resistance to standard treatments. For instance, SMO inhibitors have also shown to increase the effects of the alkylating agent temozolomide in glioblastoma xenograft models, mostly acting on the CSC population that is spared by temozolomide alone (Refs 75, 290).

Similarly, inhibition of Aurora kinase and Polo-like kinase, two important G_2_–M cell cycle regulators (Ref. 291), has shown to enhance the effect of SMO antagonist LDE-225 in blocking tumour cell proliferation *in vitro* and tumour growth *in vivo* and to increase sensitivity to conventional chemotherapy in murine *PTCH1* mutant cells and in human MB cell lines (Ref. 292).

Recently, cyclopamine has been shown to act synergistically with WIP1 inhibitor CCT007093 in reducing *in vitro* growth of patient-derived melanoma cells and BC cell lines (Ref. 215). These data suggest a possible novel therapeutic approach for tumours expressing high levels of WIP1 and with activated HH pathway, such as a subset of MB, gliomas and melanomas (Refs 215, 293, 294, 295). Targeting WIP1 in tumours with wild type p53 would lead not only to restoration of p53 tumour suppressor activity (Ref. 296), which in turn might inhibit GLI1 (Ref. 216), but also to a direct attenuation of GLI1 function (Ref. 215), resulting in a stronger inhibition of the HH pathway. This is particularly relevant to melanoma, as nearly 90% of human melanomas express functionally defective wild-type p53 and restoration of p53 function has recently been suggested as an alternative for melanoma therapy (Ref. 297). Moreover, this approach based on WIP1-p53-GLI1 axis might inhibit not only the growth of tumour bulk, but also that of putative CSCs (Ref. 215).

### Clinical trials of SMO inhibitors in combination with other targets

Based on the crosstalk between HH signalling and other pathways, several combinations with SMO inhibitors are being evaluated in clinical trials (Table 3). Most of these trials are still recruiting and do not have published data. In a clinical phase 2 study, 199 patients with metastatic colorectal cancer were treated with vismodegib or placebo in combination with VEGF inhibitor bevacizumab and chemotherapy. The study failed to show clinical benefit in vismodegib compared with placebo (Ref. 298) (Table 3). Interestingly, the placebo group had a slightly better overall response than vismodegib-treated group (51% versus 46%), probably reflecting differences in safety and tolerability, as vismodegib-chemotherapy combination is less well tolerated compared with placebo-chemotherapy combination. In a pilot study, 25 patients with metastatic pancreatic adenocarcinoma were treated with a combination of vismodegib and gemcitabine. Vismodegib treatment for 3 weeks led to downregulation of *GLI1* and *PTCH1* in post-treatment biopsies in the majority of patients, without significant changes in the CSC compartment compared with baseline. However, vismodegib and gemcitabine were not better than gemcitabine alone in the treatment of metastatic pancreatic cancer (Ref. 299).

**Table 3.** Clinical trials investigating SMO inhibitors in combination with other agents in cancer

SMO inhibitor	Combination agent^**a**^	Target	Phase	Indication	Status^**b**^	Comments	NCT # identifier^**c**^
Vismodegib (GDC-0449)	Bevacizumab, FOLFOX/FOLFIRI	VEGF	2	Metastatic colorectal cancer (*n *= 199)	Completed	ORR: vismodegib, 46% compared to placebo, 51%; common AEs: fatigue, nausea, asthenia, mucositis, peripheral sensory neuropathy, weight loss, decreased appetite, dehydration (Ref. 298)	NCT00636610
	Erlotinib, Gemcitabine	EGFR	1	Metastatic pancreatic cancer	Ongoing		NCT00878163
	Sirolimus	mTOR	1	Advanced solid tumours, including pancreatic cancer	Ongoing		NCT01537107
	RO4929097	γSecretase/Notch	1	Advanced breast cancer	Ongoing		NCT01071564
	RO4929097	γSecretase/Notch	1/2	Advanced or metastatic sarcoma	Ongoing		NCT01154452
	Leuprolide/Goserelin	GnRH agonists	1/2	Locally advanced prostate cancer	Ongoing		NCT01163084
	FOLFOX		2	Advanced gastric and gastroesophageal junction cancer	Ongoing		NCT00982592
	Gemcitabine, nab-Paclitaxel		2	Metastatic pancreatic adenocarcinoma	Recruiting		NCT01088815
	Gemcitabine		Pilot	Advanced pancreatic cancer	Ongoing	(Ref. 299)	NCT01195415
	Gemcitabine		2	Recurrent or metastatic pancreatic cancer	Ongoing		NCT01064622
	Temozolomide		1/2	Recurrent or refractory adult MB with HH pathway activation	Recruiting		NCT01601184
Sonidegib (LDE-225)	Buparlisib (BKM120)	PI3K	1	Advanced solid cancers	Recruiting		NCT01576666
	Ruxolitinib (INC424)	JAK	1/2	Myelofibrosis	Recruiting		NCT01787552
	Nilotinib	BCR-ABL	1	CML	Recruiting		NCT01456676
	Etoposide, cisplatin		1	Advanced SCLC	Recruiting		NCT01579929
	Azacitidine		1	Myeloid malignancies	Ongoing		NCT02129101
	Paclitaxel		1	Advanced solid tumours, including ovarian cancer	Recruiting		NCT01954355
	Warfarin, bupropion		1	Advanced solid tumours	Recruiting		NCT01769768
	FOLFIRINOX		1	Advanced pancreatic cancer	NA		NCT01485744
	Gemcitabine		1	Advanced pancreatic cancer	Completed		NCT01487785
	Temozolomide		2	HH-pathway activated relapsed MB	Recruiting		NCT01708174
BMS-833923	Dasatinib	BCR-ABL/Src	1/2	Chronic phase CML	Completed		NCT01218477
	Dasatinib	BCR-ABL/Src	2	Chronic phase CML	Ongoing		NCT01357655
	Cisplatin, capecitabine		1	Inoperable metastatic gastric or oesophageal cancers	Completed		NCT00909402
	Carboplatin, Etoposide		1	Extensive-stage SCLC	Completed		NCT00927875
	Lenalidomide plus dexamethasone or bortezomid		1	Relapsed or refractory multiple myeloma	Completed		NCT00884546
IPI-926	Cetuximab		1	Recurrent head and neck cancer	Completed		NCT01255800
	Gemcitabine		1/2	Metastatic pancreatic cancer	Completed		NCT01130142
PF-04449913	Ara C, decitabine, daunorubicin, cytarabine		1/2	Relapsed/refractory AML and high-risk MDS	Recruiting		NCT01546038
LY2940680	Etoposide, carboplatin		1/2	SCLC	Recruiting		NCT01722292

AEs, adverse events; AML, acute myeloid leukaemia; BCR-ABL, breakpoint cluster region-Abelson; CML, chronic myeloid leukaemia; EGFR, epidermal growth factor receptor; FOLFIRI, folinic acid, fluorouracil and irinotecan; FOLFOX, folinic acid, fluorouracil and oxaliplatin; FOLFIRINOX, folinic acid, fluorouracil, irinotecan and oxaliplatin; GnRH, gonadotropin-releasing hormone; JAK, janus kinase; mTOR, mammalian target of rapamycin; MB, medulloblastoma; MDS, myelodysplastic syndrome; ORR, overall response rate; PI3K, phosphatidylinositol-3-kinases; SCLC, small cell lung carcinoma; VEGF, vascular endothelial growth factor.

^a^Other than SMO.

^b^Status assessed on 10 December 2014; all ongoing trials are not recruiting.

^c^NCT # Identifier at http://clinicaltrials.gov.

Vismodegib is also being tested in combination with the mTOR inhibitor sirolimus, and in combination with the gonadotropin-releasing hormone agonist leuprolide or goserelin in metastatic pancreatic cancer and locally advanced prostate cancer, respectively (Table 3). In addition, clinical studies combining vismodegib with the Notch pathway inhibitor RO4929097 in advanced BC and sarcoma are ongoing. Multiple combination studies with sonidegib and BMS-833923 are either recruiting or ongoing. For instance, phase 1 studies of sonidegib in combination with PI3K inhibitor buparlisib in several types of advanced solid tumours, or in combination with BCR-ABL inhibitor nilotinib in patients with chronic myeloid leukaemia are recruiting (see Table 3 for details). Results from these clinical trials will address the applicability of SMO inhibitors in combination with other targets in multiple cancer types.

## Perspectives

Over the last decade, knowledge of the HH-GLI signalling has greatly increased, enabling a better understanding of the interaction of the major oncogenic pathways during tumourigenesis. Despite these advances, our understanding of this signalling pathway is far from complete and many important questions remain to be answered. For example, which are the mechanisms of gene regulation by GLI protein and how are cell type-specific responses determined? Are there co-factors that play a role in determining the HH transcriptional response? What is the evolutionary role of cilia in the HH signalling and do cilia play a crucial role in regulating HH signalling in human cancers?

Besides answering these questions, it will be important to develop sensitive biomarkers of HH-GLI pathway activation to identify the subset of cancers that will respond to HH inhibitors, sparing patients who are unlikely to benefit from a potentially toxic treatment. This is particularly true for MB, where only 25% harbour mutations in HH pathway genes. A reliable read-out of an active HH signalling is the expression of GLI1; however, the use of GLI1 as a biomarker by immunohistochemistry is hampered by the lack of specific GLI1 antibodies for diagnostic purposes. Equally important is to differentiate cancers with canonical and non-canonical HH pathway activation, and among the latter SMO-dependent from SMO-independent cancers. Only a clear understanding of the mechanisms leading to GLI activation in each tumour will allow for selection of the appropriate HH pathway inhibitor and, in cases where crosstalk between HH and other oncogenic pathways occurs, the optimal combinatorial partner. The prevalence of cancers with non-canonical HH activation strongly argues for the development of molecules able to target the final effectors of the HH signalling. This would provide a good approach to block ligand-independent and ligand-dependent HH pathway activation and perhaps overcome anti-SMO drug resistance.
